# Diffuse large B-cell lymphoma: the stuff of cell-of-origin and microenvironment

**DOI:** 10.18632/oncotarget.27013

**Published:** 2019-06-18

**Authors:** Stefano A. Pileri, Maria Carmela Vegliante, Sabino Ciavarella

**Affiliations:** Division of Diagnostic Hematopathology, European Institute of Oncology, IRCCS, Milan, Italy; Hematology and Cell Therapy Unit, IRCCS-National Cancer Research Center “Giovanni Paolo II”, Bari, Italy

**Keywords:** diffuse large B-cell lymphoma, cell of origin, microenvironment, prognosis, therapy

Diffuse large B-cell lymphoma (DLBCL) is the most common lymphoid malignancy. It represents a kind of Pandora’s box, containing a bulk of neoplasms which cannot be further classified by conventional histopathological criteria (DLBCLs not otherwise specified, NOS), and a series of rare entities, as primary mediastinal B-cell lymphoma or T-cell/histiocyte-rich B-cell lymphoma, provided with distinctive morpho-phenotypic features and specific therapeutic options [1]. The former group corresponds to more than 85% of all DLBCLs. R-CHOP immuno- chemotherapy cures more than 60% of patients, whereas intensification or consolidation with autologous stem cell transplantation showed no significant benefit. The remaining 35-40% of cases turns to be either refractory or relapsing, showing a dismal prognosis. Aiming to improve the outcome of these aggressive tumors, new drugs such as immunomodulators (i.e. Lenalidomide), BCR inhibitors (i.e. Ibrutinib), anti-BCL2 (Venetoclax), and immune-check point inhibitors (e.g. Nivolumab) have been proposed in first-line experimental approaches. However, they appeared expensive and so far produced results less brilliant than expected

In 2000, Alizadeh *et al*. [2] first distinguished DLBCLs/NOS into at least two main sub-groups, unrecognizable on morphologic grounds, with a gene signature related to germinal center B-cells (GCB) and activated B-lymphocytes of the peripheral blood (ABC), respectively. GCB tumors had a significantly better response to CHOP than the ABC ones, a difference that is maintained in the present immuno-chemotherapy era. Alizadeh’s and subsequent studies had the major limitation of requiring fresh/frozen (FF) samples, available only in a minimal percentage of patients. This prompted the development immunohistochemical (IHC) algorithms to surrogate gene expression profiling (GEP) on routine formalin-fixed, paraffin-embedded (FFPE) samples. However, these algorithms have provided conflicting results. In 2014, the Lymphoma Leukemia Molecular Profiling Project (LLMPP) proposed a new approach based on a targeted digital GEP, using the NanoString technology, by applying a 20-gene-panel (“Lymph2Cx”) to mRNA extracted from FFPE tissue samples [3]. Such approach produced results comparable to gold standard method and turned out superior to IHC algorithms. Consequently, the cell-of-origin (COO) determination for DLBCL/NOS was introduced as mandatory in the 2017 Revised WHO Classification of Tumors of Haematopoietic and Lymphoid Tissues. As to what the neoplastic population is concerned, recent studies of next generation sequencing (NGS) applied once again mainly to FF samples have identified novel DLBCL subgroups at least in part related to the COO (Chapuy *et al*., 2018; Schmitz *et al*., 2018). In addition, NGS remains an expensive technique requiring proper bio-informatic interpretation and producing variable results when applied to FFPE tissue.

In 2008, Lenz *et al*. [4] studied by GEP a large DLBCL set of FF samples, and firstly reported that the characteristics of tumor microenvironment (TME) represent a prognostic COO-independent factor. Two signatures were identified, namely “stromal-1” and “stromal-2”. The former, which had a prognostically favorable impact, identified tumours with brisk extracellular-matrix deposition and histiocytic infiltration. By contrast, the prognostically unfavorable “stromal-2” signature reflected higher blood-vessel density. Since then, some reports appeared in the literature aiming at translating Lenz’s signatures to FFPE tissue-samples, but all of them had no further echo.

Recently, Ciavarella *et al*. [5] generated a 1,028-gene matrix incorporating signatures of 13 immune and 4 stromal cytotypes and, using the computational method CIBERSORT [6], performed a deconvolution of a publicly available GEP dataset from 482 untreated DLBCLs. Such approach drew a map of non-malignant cellular composition, revealing unprecedented associations between clinical outcomes and proportions of putative tumor-infiltrating cytotypes. An extensive *in silico* analysis demonstrated that higher proportion of myofibroblasts (MF), dendritic cells, and CD4^+^ T-cells correlated with better outcomes, and the best 45 prognostic genes related to these cytotypes (30 MF-, 10 DC-, and 5 CD4 T cell-related genes) was selected. Their expression was measured by “NanoString” technology on a validation set of 175 pretreatment FFPE DLBCLs (staged III-IV and treated by comparable R-CHOP/R-CHOP-like regimens in two randomized trials). An unsupervised clustering analysis identified three clusters with significantly different outcome and, in a multivariate Cox model, the gene panel retained high prognostic performance, independently of COO. These data were used to build a model of clustering prediction that was successfully applied to an independent cohort of 40 “real-life” cases and, most importantly, by combining COO and TME prognosticators in a unique risk model, the Authors showed a remarkable improvement of DLBCL survival prediction.

**Figure 1:**
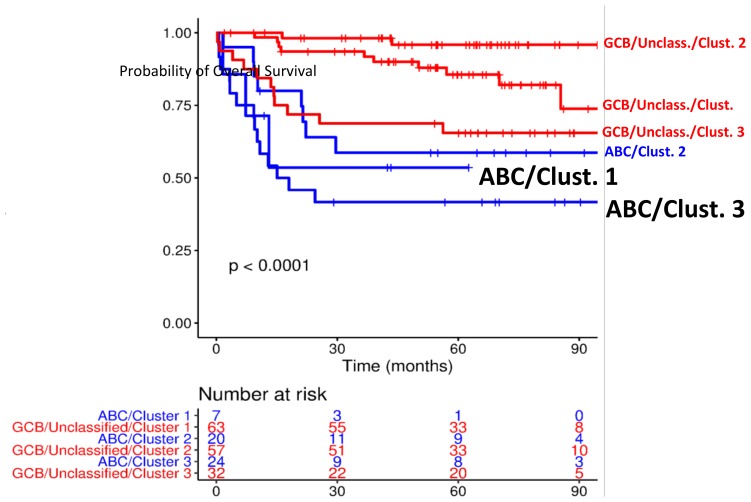
Subcategories of DLBCLs based on COO and TME profiling (Nanostring).

The work by Ciavarella *et al*. takes the concept of DLBCL heterogeneity to a new level, prompting a re-interpretation of COO categorization. Kaplan Meier curves in Figure 1 provide a representation of the capability of TME cluster assignment to split cases within either GCB/unclassified or ABC categories in smaller prognostic subgroups. Intriguingly, the subset of GCB/unclassified cases with a low-expression pattern of TME genes (cluster 3) shows poor survival, even comparable to ABC cases. Such observations claim for a deeper understanding of the biology underlying TME composition, its inter-patient variability and potential relationship with cancer genetics. In the perspective of precision medicine, individual features of TME in addition to cancer genetics could guide response prediction and optimal patient selection to novel treatments. Recent clinical trials involving DLBCL treated by R-CHOP-like regimens, especially if combined with immune/stromal modulatory drugs as Lenalidomide or Ibrutinib, should be retrospectively interpreted in the light of TME distinctions. Moreover, combined (COO+TME) profiling of DLBCLs could provide new rationales for using targeted agents with known “off-target” effects (e.g. interference of Ibrutinib on CXCR4/CXCL13 axis) [7]. Future studies could explore whether a given stromal asset may influence the effectiveness of such a drug, independently of COO. Similarly, the individual immune composition of the disease may influence the therapeutic effect of immune-modulatory compounds, especially in first-line regimens. On this respect, the Authors believe that the proposed approach would ideally fit with the emerging clinical trial designs exploring drugs for rare patient subsets [8]. New “master protocols” are expected to satisfy the need for DLBCL of a unique platform, instead of several truly independent trials, in which each case is assayed for multiple prognosticators/biomarkers and immediately assigned to an appropriate sub-study.

In conclusion, the approach proposed by Ciavarella and co-workers, based on a cheap tool easily applicable to FFPE samples, may contribute to better understand the pathobiology, chemoresistance and progression of DLBCL/NOS, as well as to design and test new therapeutic strategies.
